# Imaging Assessment of Cardiovascular Disease in Systemic Lupus Erythematosus

**DOI:** 10.1155/2012/694143

**Published:** 2011-10-23

**Authors:** Sara C. Croca, Anisur Rahman

**Affiliations:** Centre for Rheumatology, Division of Medicine, University College London, London WC1E 6JF, UK

## Abstract

Systemic lupus erythematosus is a multisystem, autoimmune disease known to be one of the strongest risk factors for atherosclerosis. Patients with SLE have an excess cardiovascular risk compared with the general population, leading to increased cardiovascular morbidity and mortality. Although the precise explanation for this is yet to be established, it seems to be associated with the presence of an accelerated atherosclerotic process, arising from the combination of traditional and lupus-specific risk factors. Moreover, cardiovascular-disease associated mortality in patients with SLE has not improved over time. One of the main reasons for this is the poor performance of standard risk stratification tools on assessing the cardiovascular risk of patients with SLE. Therefore, establishing alternative ways to identify patients at increased risk efficiently is essential. With recent developments in several imaging techniques, the ultimate goal of cardiovascular assessment will shift from assessing symptomatic patients to diagnosing early cardiovascular disease in asymptomatic patients which will hopefully help us to prevent its progression. This review will focus on the current status of the imaging tools available to assess cardiac and vascular function in patients with SLE.

## 1. Introduction

Systemic lupus erythematosus (SLE) is a multisystem, autoimmune disease and is one of the strongest known risk factors for atherosclerosis and coronary artery disease (CAD) [1, 2]. The range of cardiovascular disease (CVD) in SLE is broad and includes atherosclerosis, vascular inflammation, Raynaud's phenomenon, endothelial dysfunction, and a procoagulant tendency associated with antiphospholipid antibodies. The impact of SLE-associated CVD on both mortality and morbidity is impressive: the incidence of CAD is over 7 times greater in patients with SLE than in healthy controls, even when matched for cardiovascular risk factors [3]. Moreover, female patients with SLE between 35 and 44 years old have an incidence of myocardial infarction over 50 times greater than the observed in the Framingham dataset [4]. These findings account for the bimodal mortality pattern in SLE: an early peak (<1 year of diagnosis) associated with renal involvement and infection and a later peak (8 years after diagnosis) due to premature myocardial infarction [5]. In addition to an increased risk of CAD, patients with SLE are at greater risk for stroke, with a prevalence that can reach 20% and with a high recurrence rate and greater mortality than matched controls [6]. 

The reason why patients with SLE have a higher cardiovascular mortality and morbidity seems to be related to the presence of an accelerated atherosclerotic process [7, 8], which seems to be due to a complex interplay of traditional and lupus-specific risk factors [3, 9–11]. On the one hand, some of the factors contributing to an accelerated atherosclerosis may be associated with the disease itself: the systemic inflammation associated with poorly controlled SLE could contribute to plaque destabilization. On the other hand, patients with SLE have a high prevalence of traditional CVD risk factors [3, 10] such as hypertension, altered lipid profile [12], and impaired glucose tolerance, which to some extent result from chronic corticosteroid therapy [13]. However, not only has no unequivocal correlation been established between corticosteroid use and atherosclerosis in SLE, but some evidence seems to suggest an increased cardiovascular risk among patients who are under treated with steroids, thus implying that having poorer disease control is associated with a higher vascular risk than steroid therapy *per se* [7]. The generally accepted notion is that systemic inflammation related to SLE contributes both to an accelerated atherosclerosis and plaque destabilization which in turn is the major cause of acute plaque disruption responsible for acute cardiovascular events such as myocardial infarction.

The relevance of accurate cardiovascular assessment in patients with SLE has been emphasized by recent studies that show that mortality associated with CVD has not improved over time, opposing the trend seen for other causes of mortality such as lupus nephritis [14, 15]. Several reasons can be hypothesised to explain this but one of the strongest is the poor performance of standard risk stratification tools (based on the Framingham risk equation) [16] in patients with SLE, which prevents an accurate assessment of the actual cardiovascular risk of the individual patient [9, 17]. It is, therefore, essential to find alternative ways to assess and identify patients with SLE at increased risk for CVD efficiently. Several imaging techniques have been studied as potential tools to assess these patients better, with particular emphasis in noninvasive screening tools aimed at detecting subclinical atherosclerosis.

This paper will focus on the current status of imaging assessment of cardiac and vascular function among patients with SLE. There are two possible roles for this type of assessment. One is to identify CVD in patients with suspicious symptoms or other good reasons to suspect CVD (e.g., heavy smoking). The more challenging role is to diagnose CVD in asymptomatic patients with few or no risk factors other than SLE itself. It is important to remember that though SLE is associated with an increased relative risk of developing CVD compared to healthy controls, the absolute risk of developing CVD in an individual patient remains small. For example, in a recent multicentre study of 1249 patients recruited within 15 months of the diagnosis of SLE and followed for up to 8 years, only 74 patients developed CVD [18]. Thus, it would be difficult to justify invasive or repeated imaging to screen for CVD in the majority of patients with SLE.

## 2. Assessing Cardiac Involvement

SLE-associated cardiac involvement can be divided into 4 groups: pericarditis/pericardial effusion, valvular disease, myocardial dysfunction, and coronary-artery disease (CAD). For the purpose of this paper, we will focus only on the last two groups.

### 2.1. SLE-Associated Myocardial Dysfunction

In SLE, myocardial dysfunction may be due to several features such as CAD, valvular disease, drug-related cardiotoxicity (e.g., cyclophosphamide and chloroquine), and lupus myocarditis. After the introduction of corticosteroid therapy, the prevalence of autopsy-identified SLE-related myocarditis decreased from 50%–75% [19] to 25%–30% [20]. However, clinically evident lupus myocarditis is identified in less than 10% of patients, showing the high prevalence of subclinical disease [21]. In fact, clinical manifestations of SLE-associated myocarditis are subtle and nonspecific. The fact that systolic function is preserved until late stages of the disease accounts for the low sensitivity of echocardiographic assessment [22]. Although still considered the gold standard for pericardial and valvular evaluation [22, 23], its use in lupus myocarditis diagnosis is limited. However, it can give some indication of left ventricle diastolic dysfunction through the presence of impaired myocardial relaxation, decreased compliance, and increased filling pressure [22]. Another way to assess cardiac function is through left ventricle angiography, both by the conventional method and by using Technetium-99m myocardial perfusion imaging (SPECT), which permits accurate assessment of left ventricle volume and function [24, 25]. However, these methods have largely been replaced by MRI imaging [26].

The definite diagnosis of lupus myocarditis is histological, with typical features being interstitial oedema, focal necrosis/fibrosis, and focal or diffuse inflammatory cellular infiltrates [27]. However, despite being the gold standard for diagnosis, endomyocardial biopsy cannot be used routinely or repeatedly, particularly in asymptomatic patients. Cardiac magnetic resonance (CMR) is sensitive to many of the changes that characterize lupus myocarditis, particularly through T2-weighted imaging (myocardial oedema) [28, 29] and early (EGE) and late (LGE) gadolinium-enhanced CMR [28, 30]. The combination of EGE, LGE, and T2 imaging sequences has been reported to have 76% sensitivity and 95.5% specificity for the detection of myocardial inflammation [28]. In addition, CMR is superior to other techniques in assessment of left ventricle size, function, and mass, provides high spatial resolution, is noninvasive and has high reproducibility and low intra and interobserver variability [26]. In a recent study, we carried out CMR and transthoracic echocardiography in 22 patients with SLE (11 patients with previous CVD and 11 age-sex matched controls) [30]. We found that CMR was more sensitive than echocardiography for the detection of myocardial changes, especially late gadolinium enhancement (LGE) in areas of previous infarction [30]. In contrast to a previous report [31], we did not find widespread small areas of LGE in the myocardial tissues of these patients. Mavrogeni et al. [32] reported LGE in 18/20 patients with autoimmune rheumatic diseases (three with SLE). Ten patients also had myocardial biopsies with a 50% agreement between biopsy and CMR results.

CT imaging is not considered an adequate tool for evaluation of cardiac muscle due to radiation exposure, movement artefacts and application of contrast media which prevents use in patients with renal failure and severe heart failure [33].

### 2.2. Coronary Artery Assessment

As stated before, SLE is associated with a significantly increased risk of CAD. The presence of CAD can be evaluated directly by coronary arteriography and indirectly by assessing left ventricle ejection function and ventricular wall motion through radionuclide ventriculography, echocardiography, SPECT, and CMR [22].

2D echocardiography is the most widely used method for routine assessment of left ventricle ejection fraction in patients with known CAD. Other methods, such as tissue Doppler imaging and 3D echocardiography have been proposed as superior alternatives; however, they still have not replaced conventional echocardiography [22]. Turiel et al. [34] have proposed a global index of left ventricle function (TEI index) aimed at systolic and diastolic left ventricle function. However, its validity in SLE has yet to be shown. Stress echocardiography using either exercise or pharmacological stimulus can be a useful method for diagnosis and risk stratification in patients with suspected or known CAD [22]. 

Presently, several studies have shown the utility of MRI imaging in assessment of CAD—though not in patients with SLE [26, 28, 35]. Stress CMR (i.e., using dobutamine or adenosine) is an accurate method to identify ischemia-induced wall motion abnormalities, with a greater sensitivity (86% versus 74%) and specificity (86% versus 70%) than stress echocardiography [36]. In addition, perfusion defects can be identified with gadolinium-enhanced CMRI as well as positron emission tomography (PET) [37] and SPECT [25]. One additional benefit from PET imaging is the possibility of identifying stable plaques as a high uptake of contrast seems to be associated with a higher macrophage content which would correlate with the presence of intraplaque active inflammation [38]. However, the use of radioisotopes for PET limits its applicability.

Electron beam CT (EB-CT) can be used to quantify coronary artery calcification as a measure of coronary atherosclerosis. Asanuma et al. [7] compared EB-CT findings in 65 patients with SLE and 68 age-/sex-/ethnicity-matched controls. Mean calcification scores was significantly higher in patients than controls. After adjustment for cardiac risk factors including age, sex, smoking, hypertension, triglyceride, and homocysteine levels, patients with SLE were still 9.8 times more likely to have coronary calcification than controls. The reason for this was unclear. In a subsequent paper Kiani et al. [39] found coronary calcification in 43% of 200 women with SLE, but the only factors predicting this in multiple logistic regression analysis were age and body mass index. SLE disease activity was not associated with coronary calcification. CT angiography can be used to detect plaques in the coronary arteries. In a recent study [40], Ishimori et al. carried out both adenosine stress CMR and CTA in 18 female patients with SLE who had suffered chest pain within the previous six months and in 10 healthy control women. Eight patients with SLE, but no control subjects had abnormal perfusion on stress CMR. This was severe in 7 cases even though none of the patients had obstructive CAD detectable by CTA and only two of the 18 subjects had any CTA abnormalities. The perfusion defects were not characteristic of coronary artery disease [40]. Thus, it seems likely that stress CMR was detecting microvascular ischemia in patients with SLE though larger studies are required. 

Invasive methods for assessing coronary circulation such as intravascular ultrasound (IVUS) and IVUS with virtual histology [41], optical coherence tomography [42], coronary angioscopy [43], and invasive MRI [44] may prove their usefulness in the future by allowing direct plaque imaging. However, presently their predictive value and impact on risk stratification is yet to be established.

## 3. Assessing Peripheral Vascular Involvement

Peripheral vascular involvement in SLE can be associated with active vasculitis, endothelial dysfunction and atherosclerosis. For the purpose of this paper, we will focus on the latter two.

### 3.1. Endothelial Dysfunction

The endothelium is the main regulator of vascular wall homeostasis. It regulates vascular tone and permeability, platelet and leukocyte adhesion and aggregation, and finally, vascular thrombosis. The term “endothelial dysfunction” describes a nonadaptive state of phenotypic modulation characterized by a loss or deregulation of the homeostatic mechanisms operative in healthy endothelial cells [45]. Current evidence suggests that endothelial dysfunction is an early event in atherogenesis and contributes to all the stages of plaque development [46]. Although there are currently no imaging methods that can effectively assess endothelial function, several functional methods have been developed to try and address this issue. The hallmark of endothelial dysfunction is an impaired endothelium-dependent vasodilatation [47]. Peripheral studies include flow-mediated vasodilatation assessment [47–49], forearm perfusion techniques, pulse wave analysis, and skin laser Doppler flowmetry [45]. Other potential markers of endothelial dysfunction correlate with circulating procoagulant, prothrombotic, and proinflammatory mediators, but with the exception of C-reactive protein, evidence for their independent predictive value is still lacking [45]. Although very few studies of this nature have been done in SLE [48, 49], attenuated flow-mediated dilation has been a consistent finding, suggesting the presence of impaired endothelial function in these patients even before overt cardiovascular disease is apparent.

### 3.2. Peripheral Vascular Assessment

The presence of common carotid artery intimal-medial thickening and discrete, nonobstructive carotid atherosclerosis has been shown to be independently associated with subsequent cardiovascular risk in several longitudinal studies [50, 51].

Ultrasound assessment of carotid atherosclerosis is an accurate, noninvasive method that allows for assessment of arterial wall thickness and degree of plaque. Manzi et al. [52] studied the prevalence of carotid atherosclerosis as measured by B-mode ultrasound in 175 women with SLE, finding that 40% had at least 1 focal plaque and that more than 20% had a at least one large plaque (>50% of the vessel diameter) or multiple plaques with at least one medium plaque (30%–50% of the vessel diameter). Patients with higher cumulative damage measured by the modified Systemic Lupus International Collaborative Clinics (SLICC) damage score were more likely to have plaque, even after excluding the cardiovascular components of the SLICC index. A strong association between duration of use and cumulative dose of corticosteroids was also found. Other groups, working independently have also found a prevalence of carotid plaque in the order of 40% in patients with SLE [8]. A longitudinal study from the Manzi group [53] assessed plaque progression in 217 female patients with SLE followed for 10 years using ultrasound. Progression of plaque occurred in 27% of patients and, overall, the mean increase in intima-media thickness was 0.011 mm/year. Plaque progression was greater in patients with SLE when compared with matched controls, suggesting that B-mode ultrasound may be a useful surrogate end point in SLE clinical management [53]. Importantly, this group went on to show, for the first time, that increase in IMT or the presence of plaque predicted increased risk of cardiovascular events [54]. They followed 224 women with lupus but no previous cardiovascular events. Over a 10-year followup period, 73 of them suffered either cardiac or cerebrovascular events. In multivariable analysis, higher IMT and presence of plaque at baseline predicted increased risk of cardiovascular events. For IMT, the hazard ratio was 1.24 (95% CI 1.04 to 1.48) per mm increase and the hazard ratio for presence of plaque was 5.97 (95% CI 1.52 to 23.38) [54]. Further enhancement of ultrasound assessment of carotid plaques can be achieved using integrated backscatter analysis of carotid-intima complex. This method has been shown to correlate with the calcium and collagen content of vascular wall, therefore noninvasively evaluating arterial sclerosis [55]. However, its usefulness in SLE has not been established [56].

High-resolution CT imaging has also been studied as an alternative and more accurate method of assessing carotid plaques. CT angiography has been shown to not only provide an accurate analysis of the degree of stenosis but also to correlate with histological findings of atheromatous plaques at the carotid bifurcation [57]. However, limitations associated with the use of contrast and radiation exposure are of concern.

A potential role for MRI imaging has emerged; as its use has much less limitation than CT methods [58], and there is a good correlation between them. In addition to assessing intimal and medial thickening, both methods yield information concerning the pattern of plaque calcification. Whether this correlates with the risk of embolic stroke is yet to be definitely established.

Presently, cutting edge, multimodal imaging research using animal models is aimed at determining ways to accurately assess plaque stability [59–61]. In animal models, factors like atherosclerotic plaque neovascularisation, thickness of fibrous plaque, lipid-rich necrotic-core, and macrophage content have been related to an increased plaque disruption risk. However, clinical implications in humans have not been established.

Table 1 summarises the different methods of imaging described in this paper with their advantages and disadvantages.

## 4. Conclusion

In summary, patients with SLE have a high risk of developing CVD. Despite their relevance, traditional reversible risk factors solely cannot account for the overall cardiovascular risk increase, which also depends on disease and treatment related issues. In this paper, we have described a number of technological advances that have enhanced the ability of clinicians to assess the myocardium, coronary arteries, and peripheral vessels in patients with CVD. For most of them, there is little or no information about use in patients with SLE. Some of these imaging techniques, for example, PET scanning and CT angiography, should clearly be reserved for patients with SLE with known CVD or very high CVD risk (based on traditional risk factors as well as the presence of SLE). Others, such as echocardiography and carotid ultrasound are convenient and noninvasive and could be used as screening tools in asymptomatic patients though it is still unclear how best to manage patients who have abnormalities on these tests. Perhaps the best way to use these imaging methods in the future will be in combination with assessment of traditional risk factors, disease activity measurements, and blood tests relevant to CVD [62]. This holistic assessment could then be used to identify patients who would benefit from more accurate but more invasive imaging methods such as cardiac MRI.

## Figures and Tables

**Table 1:** Overview and comparison of different imaging methods in atherosclerotic plaque assessment (IMT: intima-media thickness; CVD: cardiovascular disease).

Imaging method	Plaque characterization	Advantages	Disadvantages	Published data from patients with SLE
Carotid ultrasound	IMT and plaque in carotid arteries	No radiation rapid-convenient correlates with risk of future CVD	Interpretation is operator dependent. High frequency of plaque in Patients with SLE (clinical implications unclear)	Yes [22, 23, 54, 55, 58]
Magnetic resonance imaging (MRI)	Structure of myocardium quantification of lipid content	No radiation more sensitive than echo for myocardial change	Expensive use of gadolinium limited in patients with renal impairment motion artefacts. Lower spatial resolution in vascular assessment. Longer length of study time	Yes [2, 13, 18, 30, 33–36]
Computed tomography (CT)	Quantification of calcium, fibrous and lipid component	Noninvasive detection of vulnerable plaques	Motion artefacts. Contraindicated in renal impairment Low resolution	Yes [17, 41, 42]
Intravascular ultrasound-based methods	Plaque volume Luminal and vessel dimensions calcium content	Good penetration depth complements coronary angiography	Invasive lower spatial resolution	No
Positron emission tomography (PET)	Plaque macrophage content		Not established for widespread clinical use	Yes [26, 27]
Optical CT	Plaque microstructure (fibrous cap thickness measurement)	High spatial resolution	Invasive limited depth of penetration	No
Invasive MR	Plaque morphology and structure		Not established for widespread clinical use	No
Coronary angioscopy	Direct plaque surface visualization	Three-dimensional view of plaque	Superficial assessment of plaque. Risk of coronary occlusion	No
